# A novel bis(pyrazolyl)methane compound as a potential agent against Gram-positive bacteria

**DOI:** 10.1038/s41598-021-95609-z

**Published:** 2021-08-11

**Authors:** Pedro Seguí, John J. Aguilera-Correa, Elena Domínguez-Jurado, Christian M. Sánchez-López, Ramón Pérez-Tanoira, Ana V. Ocaña, José A. Castro-Osma, Jaime Esteban, Antonio Marcilla, Carlos Alonso-Moreno, Francisco C. Pérez-Martínez, Milagros Molina-Alarcón

**Affiliations:** 1 Department of Otorhinolaryngology, Complejo Hospitalario Universitario, 02006 Albacete, Spain; 2 Instituto de Investigación en Discapacidades Neurológicas (IDINE), University of Castilla-La Mancha, 02001 Albacete, Spain; 3 Clinical Microbiology Department, IIS-Fundacion Jimenez Diaz-UAM, 28040 Madrid, Spain; 4 NanoCRIB Unit, Centro Regional de Investigaciones Biomédicas, 02008 Albacete, Spain; 5 School of Pharmacy, University of Castilla-La Mancha, 02008 Albacete, Spain; 6 Department of Farmàcia i Tecnologia Farmacèutica i Parasitologia, University of Valencia, Burjassot, 46100 Valencia, Spain; 7 Joint Research Unit on Endocrinology, Nutrition and Clinical Dietetics, Health Research Institute La Fe-Universitat de València, 46026 Valencia, Spain; 8 Clinical Microbiology Department, Hospital Universitario Príncipe de Asturias, Madrid, Spain; 9 Biomedicine and Biotechnology Department, School of Medicine, University of Alcalá de Henares, Madrid, Spain; 10 Department of Nursing, University of Castilla-La Mancha, 02071 Albacete, Spain

**Keywords:** Drug discovery, Microbiology, Chemistry

## Abstract

This study was designed to propose alternative therapeutic compounds to fight against bacterial pathogens. Thus, a library of nitrogen-based compounds bis(triazolyl)methane (1T–7T) and bis(pyrazolyl)methane (1P–11P) was synthesised following previously reported methodologies and their antibacterial activity was tested using the collection strains of *Staphylococcus aureus, Enterococcus faecalis*, *Escherichia coli,* and *Pseudomonas aeruginosa*. Moreover, the novel compound 2P was fully characterized by IR, UV–Vis and NMR spectroscopy. To evaluate antibacterial activity, minimal inhibitory concentrations (MICs), minimal bactericidal concentrations (MBCs), minimum biofilm inhibitory concentrations (MBICs), and minimum biofilm eradication concentrations (MBECs) assays were carried out at different concentrations (2–2000 µg/mL). The MTT assay and Resazurin viability assays were performed in both human liver carcinoma HepG2 and human colorectal adenocarcinoma Caco-2 cell lines at 48 h. Of all the synthesised compounds, 2P had an inhibitory effect on Gram-positive strains, especially against *S. aureus*. The MIC and MBC of 2P were 62.5 and 2000 µg/mL against *S. aureus*, and 250 and 2000 µg/mL against *E. faecalis*, respectively. However, these values were > 2000 µg/mL against *E. coli* and *P. aeruginosa*. In addition, the MBICs and MBECs of 2P against *S. aureus* were 125 and > 2000 µg/mL, respectively, whereas these values were > 2000 µg/mL against *E. faecalis*, *E. coli*, and *P. aeruginosa*. On the other hand, concentrations up to 250 µg/mL of 2P were non-toxic doses for eukaryotic cell cultures. Thus, according to the obtained results, the 2P nitrogen-based compound showed a promising anti-Gram-positive effect (especially against *S. aureus*) both on planktonic state and biofilm, at non-toxic concentrations.

## Introduction

Despite of several emerging antibiotic families effective against pathogenic bacteria existing^1,2^, strains resistant to current therapeutic options have become one of the most important challenges in this field. Indeed, antibiotic-resistant strains have been identified between both Gram-positive and Gram-negative bacteria^3,4^ with extraordinarily high mortality and morbidity rates worldwide^1,5^. To date, these infections are treated with different combinations of antibiotics, but they can produce severe undesired side effects^6^.

Antibiotic resistances are considered a worrying economic burden that leads to an estimated extra annual cost of $2.2 billion in the United States alone^7^. The risk and cost related to bacterial resistance to chemical products have drawn interest in discovering new compounds with antimicrobial effects^8,9^. In this field, the chemistry of metallic ions, enzybiotics, natural extracts or new nanotechnology-based delivery systems are leading research lines^10,11^.

On other hand, bacteria can exist in two non-excluding lifestyles: planktonic or free-life form, or in a sessile form named biofilm. A biofilm is a bacterial aggregate embedded in a self-produced exopolymeric matrix where numerous and complex sociomicrobiological interactions rule^12^. Biofilm of a bacterium exhibits different inherent characteristics that confer it resistance to almost any unfavorable condition (e.g. the attack of the immune system) and antibacterial compounds, such as antibiotics, reactive oxygen species, and heavy metals. Therefore, if the growth of any bacterium is inhibited or completely prevented, the development of the biofilm of such bacterium is also prevented^13^. In this regard and with the aim to prevent antibiofilm formation, the development of quorum sensing inhibitors have been one of the most promising strategy^14–16^.

Bis(triazolyl)methane and bis(pyrazolyl)methane-based nitrogen compounds have been previously used as auxiliary tridentate ligands for the synthesis of organometallic compounds^17^. These counterparts have been extensively employed as catalysts for polymerisation, epoxidation and oxidation processes^18^. From a chemical point of view, the comparatively rapid and modular synthesis of bis(triazolyl)methane^19^ and bis(pyrazoyl)methane^20^ nitrogen-based scaffolds present an ideal template for both high-throughput and rational drug design. Moreover, the potential utility of this family of compounds as an easily tunable core in both rapid drug discovery and subsequent logical designs of new antibacterial metal-containing drugs remains unknown^21^.

This study was conducted to screen bis(triazolyl)methane and bis(pyrazolyl)methane derivatives as compounds with antibacterial activity against both planktonic and biofilm states.

## Results

### Synthesis and characterisation of the bis(triazolyl)methane and bis(pyrazolyl)methane derivatives

**Figure 1:**
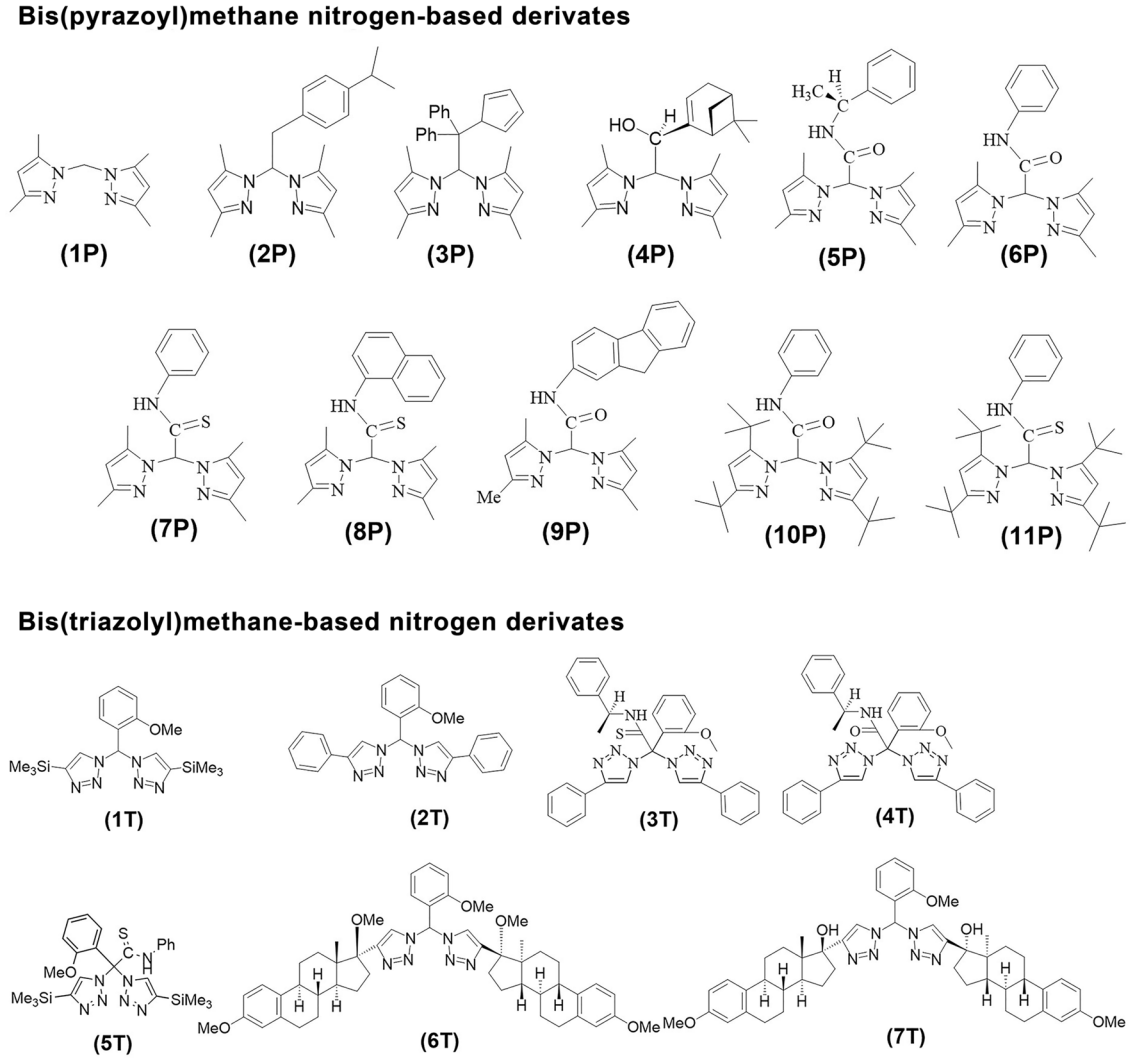
Chemical structures of both series of compounds.

**Figure 2:**
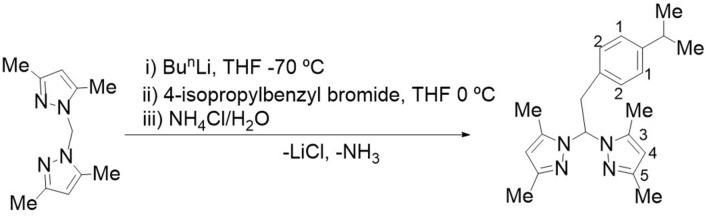
Synthetic route for compound 2P.

Most of the compounds herein used were synthesised according to previously published procedures (see the chemical structures in Fig. 1)^19,20,22^. Briefly, 1T–7T were obtained by reaction between readily accessible alkynes and geminal diazides in a double Cu-catalyzed azide-alkyne cycloaddition reaction. The compounds 1P–11P were previously obtained from the corresponding lithium salts and subsequent hydrolysis by saturated aqueous ammonium chloride solutions. In particular, the novel compound 2P was obtained as a pale yellow solid by one-pot reaction of bis(3,5-dimethylpyrazol-1-yl)methane with nBuLi, followed by the addition of 4-isopropylbenzyl bromide to THF at 0 °C (Fig. 2). The compounds 1T–7T and 1P–11P were not soluble in water but air stable and soluble in chlorinated solvents. Qualitative tests determined that the compounds are soluble in polar protic solvents such as methanol, and ethanol, and polar aprotic solvents such as dimethyl sulfoxide and tetrahydrofuran.

**Figure 3:**
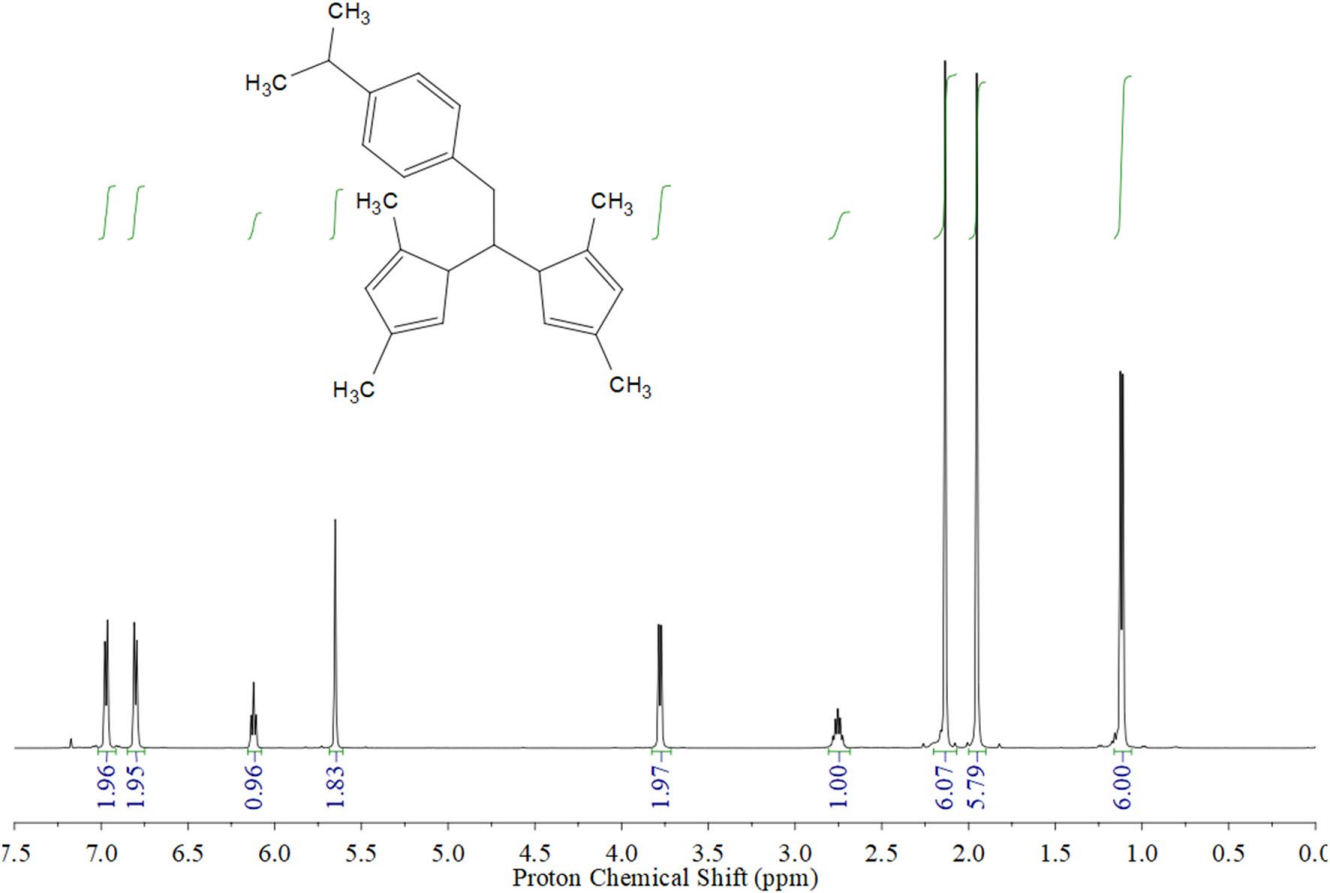
^1^H-NMR spectrum for the 2P compound in CDCl_3_ at 297 K.

The 2P nitrogen-based compound characterisation was carried out by IR, UV and NMR spectroscopy (Fig. 3 and Fig. S1–S4 in the Supporting Information). Structural elucidation is depicted in the “Methods” section and illustrated by the ^1^H-NMR spectrum in Fig. 3. In the IR spectrum of 2P (solid state), the C=N functional group was depicted by a broad band at 1378 cm^−1^, while the strong absorption at 1019 cm^−1^ was attributed to the in-plane bending vibration of pyrazole moiety. The ^1^H and ^13^C-{^1^H} NMR spectra of 2P contained a single set of resonances for pyrazole rings, which indicates that the two pyrazole rings were equivalents. The ^1^H–^13^C heteronuclear correlation (g-HSQC) experiments enabled us to assign the resonances corresponding to C^4^, Me^3^ and Me^5^ of the pyrazole ring (see “Methods” and Fig. S2 in the Supporting Information). The presence of signals at 1.12 and 2.70 ppm and at 24 and 33 ppm in the ^1^H and ^13^C{^1^H} NMR spectra of 2P, respectively, confirmed that isopropylbenzyl moiety was present in the compound.

### Antibacterial assessments

**Figure 4:**
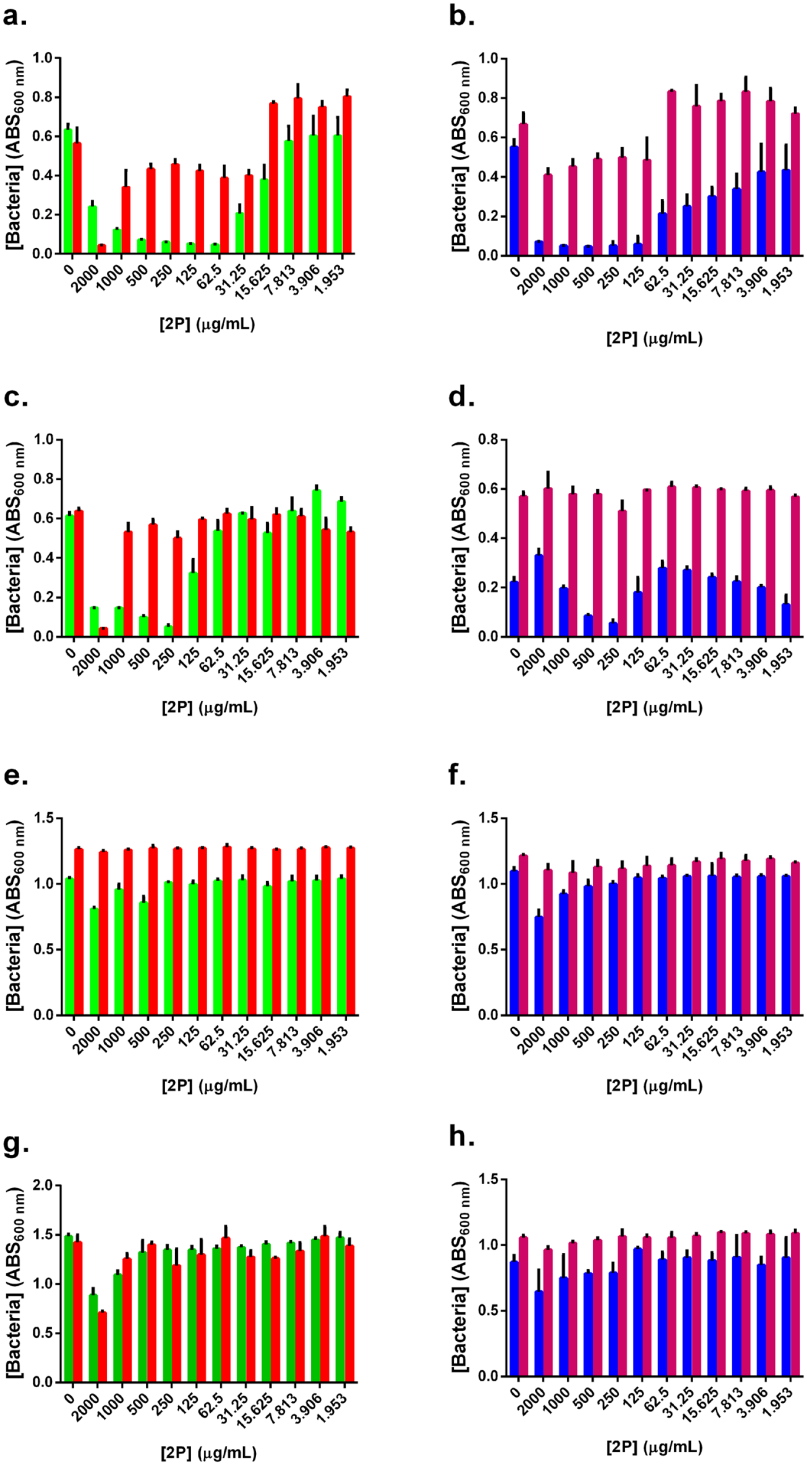
Minimal inhibitory concentrations (MICs, green) and minimal bactericidal concentrations (MBCs, red) (**a**,**c**,**e**,**g**), as well as minimal biofilm inhibitory concentrations (MBICs, blue) and minimal biofilm eradication concentrations (MBECs, magenta) (**b**,**d**,**f**,**h**) of 2P against *S. aureus* (**a**,**b**), *E. faecalis* (**c**,**d**), *E. coli* (**e**,**f**), and *P. aeruginosa* (**g**,**h**). Bars represent the interquartile range. *ABS* absorbance.

The antibacterial effect of the compounds was evaluated by studying both MICs and MBCs. For this purpose, the different compounds were studied against planktonic bacteria of *S. aureus, E. faecalis, E. coli,* and *P. aeruginosa at* different concentrations. Except for the 2P compound, none of the compounds showed any activity against the studied bacteria. The MIC and MBC of 2P were found to be 62.5 and 2000 µg/mL against *S. aureus* (Fig. 4a), and 250 and 2000 µg/mL against *E. faecalis* (Fig. 4c), respectively. The MICs and MBCs of 2P against *E. coli* (Fig. 4e) and *P. aeruginosa* (Fig. 4g) were > 2000 µg/mL for both of them.

In addition, the antibiofilm effect of compounds was evaluated by studying both MBICs and MBECs. For this purpose, the different compounds were tested against biofilms *S. aureus, E. faecalis, E. coli,* and *P. aeruginosa* at different concentrations. As observed against the planktonic bacterial strains, only the 2P derivative had antibiofilm effects. Thus the MBICs and MBECs of 2P against *S. aureus* were 125 and > 2000 µg/mL, respectively (Fig. 4b). The MBICs and MBECs of 2P against *E. faecalis* (Fig. 4d), *E. coli* (Fig. 4f) and *P. aeruginosa* (Fig. 4h) were > 2000 µg/mL for both of them (Fig. 4b). However, *E. coli* and *P. aeruginosa* showed a slight, but significant, drop in the bacterial concentration in MICs and MBICs at 2000 µg/mL.

### Cytotoxicity of the 2P derivative

**Figure 5:**
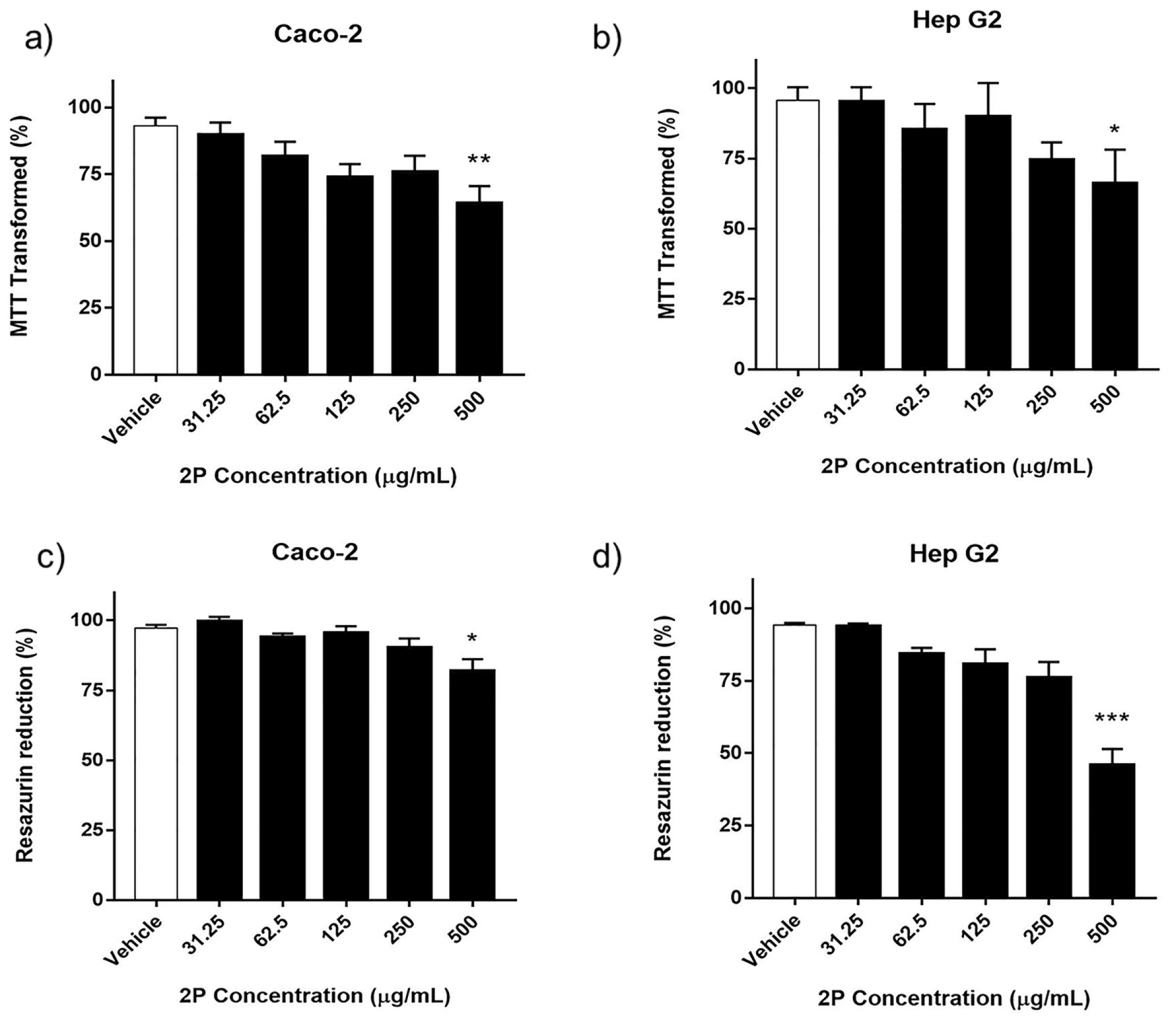
Evaluation of cytotoxicity induced by the 2P nitrogen-based compound. Cytotoxicity was determined by either the percentage of (**a**,**b**) MTT transformed or (**c**,**d**) Resazurin reduction detected in the HepG2 and Caco-2 cells exposed to concentrations from 500 to 31.25 µg/mL of the 2P derivative in MTT and Resazurin assays. The 2P compound reduced cell viability in a concentration-dependent manner, with no cytotoxicity detected up to 500 µg/mL compared to the untreated control cells. Data are expressed as mean ± SEM (n = 5). *p < 0.05; **p < 0.01; ***p < 0.001 vs. the vehicle-treated cells (using Kruskal–Wallis followed by Dunn's test).

In order to examine the toxicity caused by the 2P compound on the human liver carcinoma Hep G2 and human colorectal adenocarcinoma Caco-2 cell lines, the MTT assay or Resazurin viability assays were performed. The cell viability of Hep G2 cells at the 500, 250, 125, 62.5 and 31.25 µg/mL concentrations of 2P was 45.82 ± 12.57, 76 ± 12.3, 80.71 ± 11.55, 84.15 ± 5.03 and 93.68 ± 2.31, respectively, in the Resazurin assays, and 66.07 ± 26.95, 74.54 ± 13.73, 89.91 ± 26.55, 85.28 ± 20.31 and 95.13 ± 11.64 in the MTT assay (Fig. 5). The cell viability of Caco-2 cells was 81.89 ± 9.54, 90.29 ± 7.28, 95.41 ± 5.54, 94.06 ± 2.69 and 99.57 ± 3.83 in the Resazurin assay, and 63.98 ± 14.88, 75.79 ± 13.64, 73.83 ± 11.25, 81.68 ± 12.35 and 89.6 ± 10.71 in the MTT assay, respectively (Fig. 5).

The results showed that the studied derivative with bactericidal properties (2P) reduced the cell viability in both cell lines in a concentration-dependent manner, and no significant reduction in cell viability was observed up to 500 µg/mL of 2P compared to the untreated control cells (Fig. 5). The cytotoxic 2P concentrations (500 µg/mL) led to changes in cell morphology, as observed by microscopic observations (Fig. S3 in the Supporting Information). Our results revealed that Hep G2 cells seemed to be more susceptible than Caco-2 cells to high 2P derivative concentrations.

### pH measurements

In our study, the 2P compound induced a slight culture media alkalinisation in a time-course manner. Thus, in contrast to the results obtained with vehicle, the presence of 2P at a high dose (500 µg/mL) in the culture medium increased the pH from 7.50 ± 0.03 at 0 h to 7.62 ± 0.01, 7.82 ± 0.01, 7.99 ± 0.02, and 8,12 ± 0.01 at 6 h, 24 h, 48 h, and 72 h of incubation, respectively. This increment was statistically significant only after 48 h and 72 h of incubation, compared to vehicle-treated media (P = 0.049 and 0.037, respectively; Mann–Whitney test).

## Discussion

As resistance to antimicrobial agents by pathogenic bacteria has emerged in recent years, new compounds with antimicrobial effects have come to the forefront^9,23^. The main aim of the present study was to assess the antimicrobial activity of a series of bis(triazolyl)methane and bis(pyrazolyl)methane nitrogen-based compounds. After evaluating 18 different compounds, 2P was identified as a new derivative with significant activity against bacterial growth and/or biofilm formation at non-toxic doses for eukaryotic cells in culture. This compound and its full characterisation are herein reported for the first time.

Compound 2P displayed the ability to inhibit the bacterial growth of both *S. aureus* at 62.5 µg/mL and *E. faecalis* at 250 µg/mL, which are two clinically important Gram-positive bacteria. 2P also reduced *S. aureus* biofilm formation, and the MICs and MBICs of *S. aureus* were lower than the cytotoxic 2P concentration studied by MTT and Resazurin viability assays in both human liver carcinoma Hep G2 and human colorectal adenocarcinoma Caco-2 cell lines. The mechanism of the antimicrobial effect shown by 2P on Gram-positive bacteria is unclear, but its antimicrobial properties are probably based on several factors, including high pH and osmotic effects caused by the non-physiological concentration of dissolved ions, as observed with other compounds^24–26^. However, at high doses, a lytic effect on bacteria was also observed when the 2P nitrogen-based compound was used.

*S. aureus* as a type of Gram-positive bacterium was analyzed because it is one of the most frequent pathogens worldwide^27,28^. *E. coli* is a Gram-negative bacterium that usually affects older patients, and is the most frequent Gram-negative microorganism related to pathogenic infections^29,30^. Finally, *E. faecalis* and *P. aeruginosa* are clinically relevant Gram-positive and Gram-negative bacteria, respectively, whose presence is associated with infections in many patients^28^.

It is the first time that the antibacterial effects of 2P are reported, with MICs ranging from 125 μg/mL to over 1 mg/mL. Depending on the microbial strain and type of antimicrobial assay followed. 2P was generally more effective against Gram-positive than Gram-negative bacteria, perhaps due to differences in bacterial cell wall composition. Indeed, the 2P compound had a stronger effect against *S. aureus* Gram-positive surfaces, whose walls exhibited more negative charges than the other studied bacterial strains*.* However, other mechanisms cannot be ruled out. Recently, there was a report about several compounds possibly inactivating bacterial growth through multitarget effects due to cell membrane disruption and subsequent cell lysis, and also due to respiratory activity inhibition^31^. Besides, as the repair of sublethal injuries is associated with high energy production to regain the repair membrane damage ability^32^, the continuous depletion of the energy pool could also be responsible for 2P antibacterial effects on Gram-positive bacteria.

The generally good efficacy of the 2P compound against Gram-positive bacteria (*S. aureus* and *E. faecalis*) compared to Gram-negative ones (*E. coli* and *P. aeruginosa*) could be especially relevant because of the differences in membrane structure and peptidoglycan layer thickness^30,33^. The peptidoglycan layer of the membranes of Gram-positive bacteria (about 20–80 nm) is usually thicker than that of Gram-negative bacteria (about 7–8 nm)^34^, which makes it more difficult to penetrate and, thus, new compounds like 2P that can properly kill this kind of bacteria are very interesting. The thicker *S. aureus* membrane structure may hinder most of the compounds penetrating to the cell wall, which would reduce its toxic effects on such bacteria^35^. However, the 2P derivative could bind to Gram-positive bacteria cell walls and cause membrane disruption through direct interactions or by a lytic effect, which occurred when 2P was applied at high concentrations. This action mechanism should be less toxic in mammalian cells because these cells can degrade chemical ligands by lysosomal fusion to reduce toxicity and free radical damage^36^. In addition, other factors related to the antibacterial effects of the 2P compound on Gram-positive bacteria could be the increased osmotic pressure caused by ions that could be released from 2P, which would perturb the membrane potential of bacteria^26^, although this action mechanism should be analyzed in more depth.

2P also had inhibitory effects on *S. aureus* biofilm development, as we noted in the MBIC and MBEC assays. Although one of the implicated mechanisms has to be due to the antimicrobial activity against planktonic cells, the *S. aureus* antibiofilm effect could also result from the modification to pH during the incubation time with the 2P compound. Even though *S. aureus* is able to grow in a range of pH 4.0–9.8, with an optimum pH of 6–7, an alkaline environment is not well tolerated by microorganisms^37^ and could interfere with the *S. aureus* biofilm by reducing it or inhibiting its growth^38^.

This study firstly showed the effect of the 2P nitrogen-based compound on Gram-positive bacterial strains. However, further experiments should be performed to evaluate whether this compound had a straight effect on bacterial adhesion mechanisms or only on bacterial viability. Other experiments should also be run to evaluate 2P activity using clinical strains isolated from patients because they often show different properties compared to laboratory-adapted collection strains^39^.

## Conclusion

Pyrazoline derivatives have been widely used as pharmacophores in medicinal chemistry. In this very context, some studies confirmed their antimicrobial activity^40^. On the other hand, triazole derivatives emerged for the treatment of many systemic mycoses^41,42^. Of note, fluconazole as a triazole fungistatic agent. Based on those structures, a family of pyrazole and triazole-core compounds were synthetized and their antibacterial activity was tested against strains of *S. aureus*, *E. faecalis*, *E. coli*, and *P. aeruginosa*. Considering all the results obtained from antimicrobial screen, the 2P nitrogen-based compound is identified as a derivative with anti-Gram-positive activity and low toxicity to eukaryotic cells. Effects include an inhibitory effect on bacterial growth and biofilm formation, mainly on *S. aureus*. Based on the compounds structure, it seems that the p-cymene substitution on the pyrazole core has made a crucial contribution to the antimicrobial activity in this series of pyrazole derivatives 1P-11P. In fact, p-cymene is a monoterpene that shows a range of biological activity including antioxidant, anti-inflammatory, antinociceptive, anxiolytic, anticancer and antimicrobial effects^24^. Further studies with clinical isolates are necessary to show if 2P is a suitable candidate to develop new agents with antibacterial properties.

## Methods

### Chemicals

All manipulations were performed under nitrogen by standard Schlenk techniques^43^. THF was pre-dried over sodium wire and distilled under nitrogen from sodium. CDCl_3_ was stored over activated 4 Å molecular sieves and degassed by several freeze–thaw cycles. All the NMR experiments were conducted in deuterated solvents at 297 K in a Varian FT-400 spectrometer. The ^1^H π/2 pulse length was adjusted per sample. The ^1^H- and ^13^C{^1^H}-NMR chemical shifts (δ) were expressed as ppm in relation to TMS. Coupling constants (J) were documented in Hz. Two-dimensional NMR spectra were acquired with standard VARIAN-FT software and processed on an IPC-Sun computer. The solvent signals were used as references and chemical shifts were converted into the TMS scale. Melting points (m.p.) were determined using a melting point block (SMP 10). The sample was heated to 100 °C and then heated at a rate of 1 °C/min to 275 °C. The IR experiments were conducted on FT/IR-4000 Series Jasco Instruments. The UV–Vis absorption spectra were recorded at room temperature by a Cary 100 (Varian) spectrophotometer using a slit width of 0.4 nm and a scan rate of 600 nm/min.

### Synthesis of bis(triazolyl)methane and bis(pyrazolyl)methane-based nitrogen compounds

Bis(Triazolyl)methane derivatives (1T–7T) were prepared by a reaction between alkynes and geminal dizides in double-coupled Cu-catalysed azide-alkyne cycloaddition reactions as previously reported^19^. Compound bis(3,5-dimethylpyrazol-1-yl)methane (1P) was prepared according to procedures reported in the literature^44^. Bis(pyrazolyl)methane derivatives (3P–11P) were obtained by a one-pot reaction of bis(3,5-dimethylpyrazol-1-yl)methane (bdmpzm) or bis(3,5-di-tert-butylpyrazol-1-yl)methane with Bu^n^Li, followed by the addition of the corresponding isocyanates or isothiocyanates to provide the desired compounds^22^.

For the 2P compound synthesis, bis(3,5-dimethylpyrazol-1-yl)methane (2.00 g, 9.77 mmol) was dissolved in dry THF (50 mL) in a 250 mL Schlenk tube and cooled to − 78 °C. Then 1.6 M solution of Bu^n^Li (6.11 mL, 9.77 mmol) in hexane was added dropwise and the mixture was stirred for 1 h in a nitrogen atmosphere. The resulting mixture was transferred dropwise to a cooled (− 10 °C) solution of 4-isopropylbenzyl bromide (1.63 mL, 9.77 mmol) in THF (10 mL). Next the reaction mixture was allowed to warm to ambient temperature and stirred for 1 h. The product was hydrolysed with saturated aqueous NH_4_Cl (15 mL). The organic layer was extracted, dried over MgSO_4_ and filtered, and the solvent was removed in a vacuum to give rise to the product as an orange oil. The final product was obtained after purification by silica gel column chromatography using hexane:ethyl acetate 6:1. Yield: 45% (1.47 mg). ^1^H NMR (400 MHz, CDCl_3_, 297 K), δ (ppm): 6.97 (d, *J* = 7.5 Hz, 2H, *H*^*1*^ 4-isopropylbenzyl), 6.80 (d, *J* = 7.5 Hz, 2H, *H*^*2*^ 4-isopropylbenzyl), 6.12 (t, *J* = 7.1 Hz, 1H, C***H***-CH_2_), 5.65 (s, 2H, *H*^*4*^ pyrazol), 3.78 (d, *J* = 7.1 Hz, 2H, CH-C***H***_***2***_), 2.70 (m, 1H, C***H***-(CH_3_)_2_), 2.14 (s, 6H, C***H***_***3***_ pyrazol), 1.95 (s, 6H, C***H***_***3***_ pyrazol), 1.12 (d, *J* = 6.9 Hz, 6H, CH*-(*C***H***_***3***_)_2_). ^13^C{^1^H}-NMR (101 MHz, CDCl_3_, 297 K), δ (ppm): 147.76, 139.58 (2C, quaternary pyrazol), 147.48, 133.93 (2C, quaternary 4-isopropylbenzyl), 129.25 (2C, C^1^ 4-isopropylbenzyl), 126.39 (2C, C^2^ 4-isopropylbenzyl), 106.36 (2C, C^*4*^ pyrazol), 72.57 (1C, **C**H-CH_2_), 39.51 (1C, CH-***C***H_2_), 33.72 (1C, ***C***H-(CH_3_)_2_), 24.00 (2C, CH-***C***H_3_), 13.71 (2C, ***C***H_3_ pyrazol), 10.94 (2C, ***C***H_3_ pyrazol). IR Neat: ν_max_ 2960.021 cm^−1^ (C-H sp^3^), 1556.294 (C=C), 1511.367–1417.038 cm^−1^ (two bands, C–H aromatic), 1378.809 cm^−1^ (C=N); m.p: 356.15–358.15 K.

### Bacterial culture

Strains *S. aureus* ATCC25923, *E. faecalis* ATCC29212, *E. coli* ATCC25922, and *P. aeruginosa* ATCC27853 were used in all the microbiological studies. Unless otherwise stated, all the strains were cultured and maintained in Müller–Hinton broth solidified with 1.5% agar. Before performing the antimicrobial activity assays, each bacterial strain was refreshed in 5 mL of Müller–Hinton broth separately under sterile conditions. Strains were cultured overnight at 37 °C with 150 rpm agitation in a shaking incubator. Each bacterial culture was maintained at a concentration of 10^8^ colony forming units (CFUs)/mL.

### Determination of minimal inhibitory concentrations and minimal bactericidal concentrations

Minimum inhibitory concentrations (MICs) were determined using the previously reported broth microdilution method^45^. Briefly, a series of compounds concentrations starting from 2 to 1953 µg/mL at the two-fold dilution were added to the cation-adjusted Müller–Hinton broth (Sigma Aldrich, USA) (CAMHB) at a final volume of 100 μL per well. One hundred microlitres of bacterial suspension in CAMHB containing approximately 1.6 × 10^6^ CFU/mL were added to a Costar 96-well round-bottom polypropylene plate (Corning Inc., USA), followed by static incubation at 37 °C and 5% CO_2_ for at least 20 h. After incubation, MICs were determined by measuring absorbance at 600 nm. A series of compound concentrations at the two-fold dilution and without bacteria was used as a negative control of absorbance. Minimum bactericidal concentrations (MBCs) were determined by the flash microbiocidal method described elsewhere^46^. Briefly after 24 h incubation, 10 μL of each well were mixed with 190 μL of tryptic soya broth (Biomérieux, France) in a new 96-well plate, which was incubated statically at 37 °C and 5% CO_2_ for 24 h. After incubation, MBCs were determined by measuring absorbance at 600 nm. These experiments were performed 4 times.

### Minimal biofilm inhibitory concentrations and minimal biofilm eradication concentrations

Minimal biofilm inhibitory concentrations (MBIC) and minimal biofilm eradication concentrations (MBEC) were determined by the methodology described elsewhere^47^. For MBICs, biofilm formation on pegs from the Calgary device was induced by inoculating 200 µL of tryptic soya broth containing 10^6^ CFU/mL per well in a 96-well flat-bottom plate (ThermoFisher Scientific, USA). Then the Calgary device lid (ThermoFisher Scientific, USA) was placed and the plate was incubated in turmoil at 37 °C and 5% CO_2_ for 24 h. After incubation, the pegs from the lid were rinsed twice in the wells containing 200 µL of phosphate buffered saline (PBS). Then the lid was placed in a plate at different compound concentrations starting from 2 mg/mL to 1953 µg/mL at the two-fold dilution to be added to CAMHB at a final volume of 200 μL per well. It was incubated by static incubation at 37 °C in 5% CO_2_ for 24 h.

After incubation, MBICs were determined by measuring absorbance at 600 nm. A series of compound concentrations at the two-fold dilution and without bacteria was used as a negative control of absorbance. For MBECs, the lid from the MBIC was rinsed twice in a plate with wells containing 200 µL of PBS, placed in a plate with 200 µL of tryptic soya broth, and incubated by static incubation at 37 °C and 5% CO_2_ for 24 h. After incubation, MBECs were determined by measuring absorbance at 600 nm. These experiments were performed 4 times.

### Cell line and culture conditions

Human liver carcinoma HepG2 (ATCC^®^ HB-8065™) and human colorectal adenocarcinoma Caco-2 (ATCC^®^ HTB-37™) cell lines were grown according to the standard protocol using DMEM 4.5 g/L of glucose medium supplemented with 10% foetal bovine serum (FBS), penicillin and streptomycin (100 µg/mL) (all from Gibco™, Fischer Scientific), and cultured at 37 °C in 5% CO_2_.

### Cell viability assays

Cells were plated in 96-well culture plates and cultured at 37 °C in 5% CO_2_ for 24 h. Then cells were washed twice with PBS, and 100 µL of DMEM medium supplemented with 10% FBS, penicillin and streptomycin (100 µg/mL), were added to wells. Cells were treated with 500, 250, 125, 62.5 or 31.25 µg/mL of the 2P nitrogen-based compound, and incubated at 37 °C in 5% CO_2_ for 48 h. To determine toxicity by the 3-[4,5-dimethylthiazol-2-yl]-2,5-diphenyl tetrazolium bromide (MTT) assay, 10 µL of MTT solution (Biotium, Quimigen) were added to each well following the manufacturer instructions^48^. Briefly, cells were incubated for 4 h and then 200 µL of DMSO were added to each well. Absorbance was measured at 570 nm and 630 nm (background) in an iMark™ Microplate Absorbance Reader (Bio-Rad) by subtracting background absorbance from signal absorbance to obtain normalised values. For the Resazurin viability assays, 10 µL of Resazurin solution (Canvax Biotech) were added to each well by incubating cells for another 4-h period. Next absorbance was measured at 545 and 620 nm (background) in a Multiescan EX Microplate Absorbance Reader (ThermoFisher Scientific) by subtracting background absorbance from signal absorbance to obtain normalised values. Photographs were taken on days 1 and 3 using a Zeiss Primovert inverted microscope with an Axiocam 208 colour camera at the 100-fold magnification.

### pH study

Firstly, 250 µL of the vehicle (Milli-Q Water) or 2P compound were added to 5 mL of DMEM medium (pH 7.4) to reach a final concentration of 0 or 500 µg/mL of the 2P compound, respectively. Then the treated mediums were incubated at 37 °C in 5% CO_2_, and the pH of solutions was measured at 0, 6, 24, 48 and 72 h using a pH 8+ DHS Stirrer pH meter (XS instruments). The pH studies were done in triplicate.

### Statistical analysis

Statistical analysis was performed using the SPSS 13.0 (SPSS, Chicago, IL, USA). Mann–Whitney test or a non-parametric variance analysis (Kruskal–Wallis) followed by Dunn's test were used for the statistical analysis.

## Supplementary Information

Supplementary Figures.
