# Supplementary material for: A novel bis(pyrazolyl)methane compound as a potential agent against Gram-positive bacteria
Source: Sci Rep. 2021 Aug 11;11:16306. doi: 10.1038/s41598-021-95609-z (PMC8357914; doi:10.1038/s41598-021-95609-z)

**Supplementary material**

**Suppl Figure 1** ^13^C{^1^H}-NMR spectrum for the 2P derivative in CDCl_3_ at 297K.

**Suppl Figure 2** HSQC spectrum for the 2P derivative in CDCl_3_ at 297K.

**Suppl Figure 3** IR spectrum for the 2P derivative.

**Suppl Figure 4** UV/Vis absorbance spectrum for the 2P derivative in THF.

**Suppl Figure 5** Morphology of the Hep G2 (a; b) and Caco-2 (c; d) cells in culture when treated with different compound 2P concentrations. Light microscopy observations showed that 500 µg/mL of 2P induced changes in the shape of the HepG2 (b) and Caco-2 (d) cells, whereas the use of 31.25 µg/mL of 2P did not affect the morphology of HepG2 (a) and Caco-2 (c).

**Suppl. Figure 1**


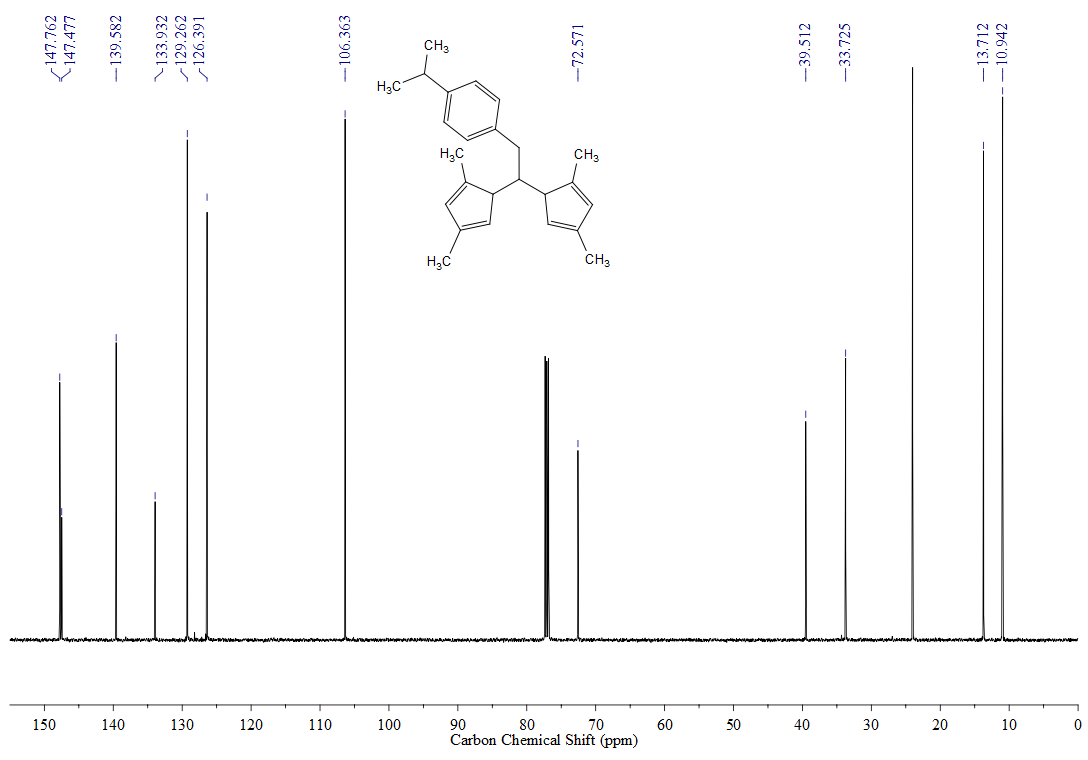


**Suppl. Figure 2**


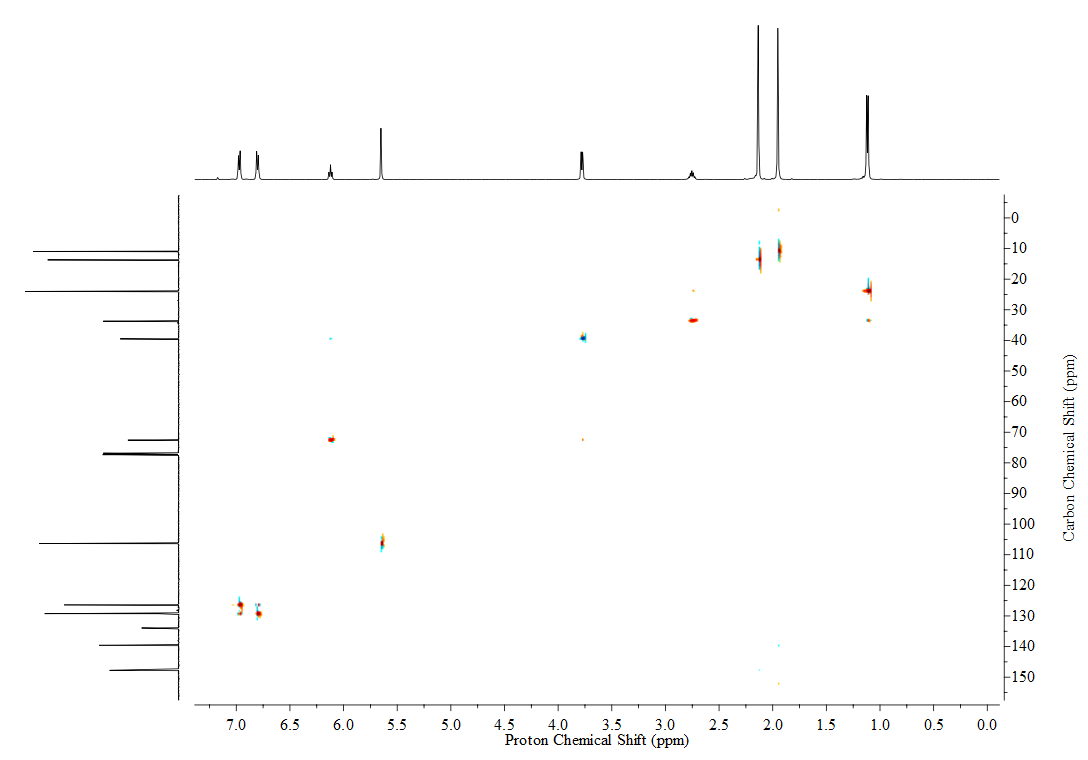


**Suppl. Figure 3**


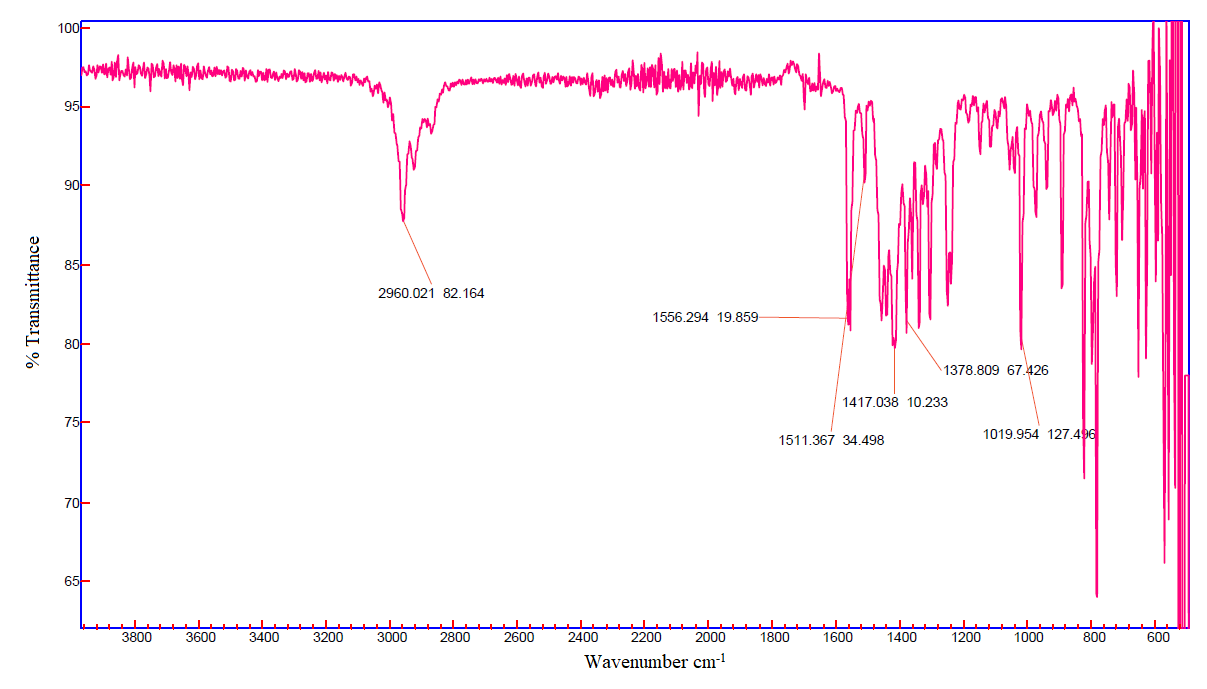


**Suppl. Figure 4**

**Suppl. Figure 5**


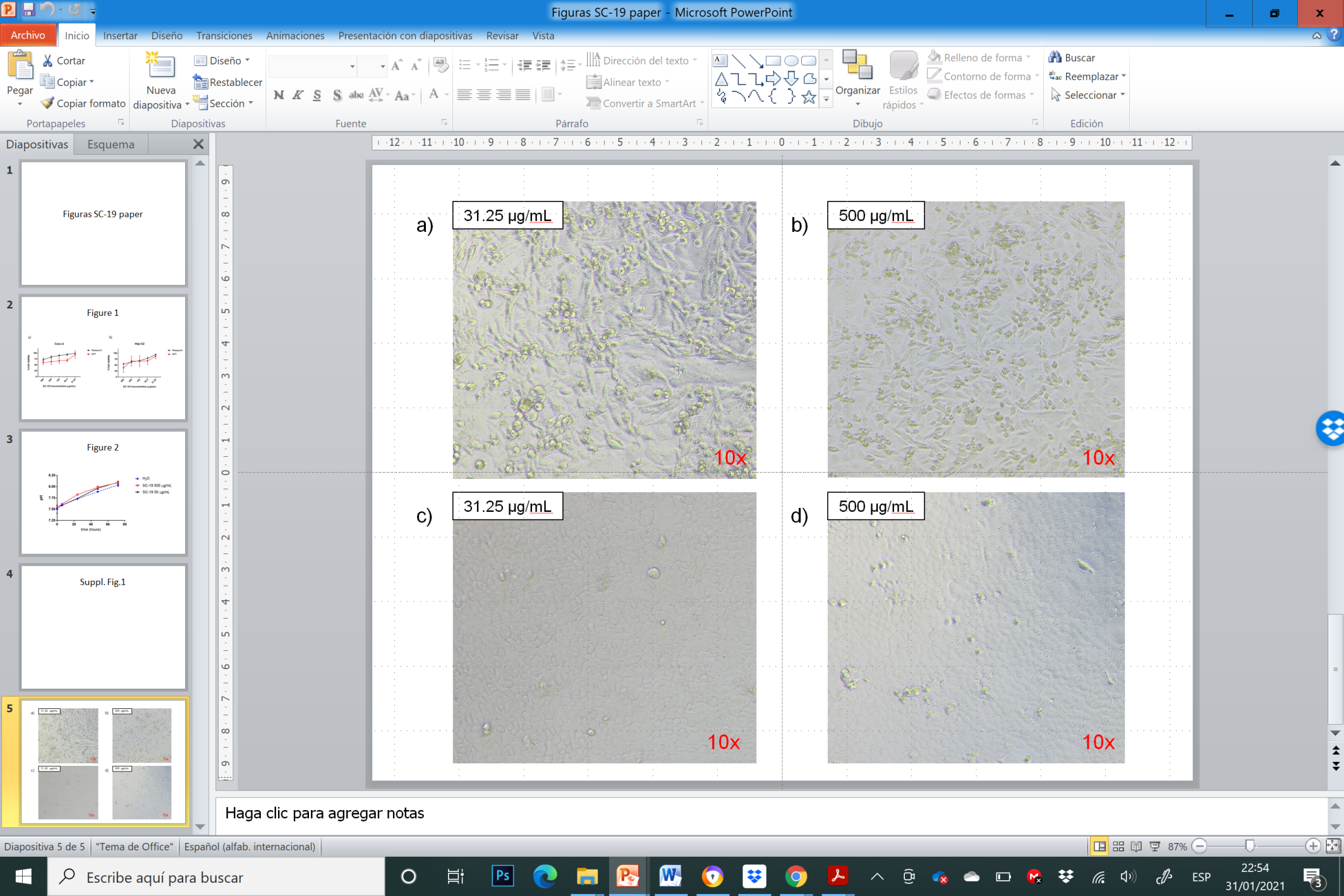

Supplement: Supplementary file 1 — Supplementary Figures. [file 41598_2021_95609_MOESM1_ESM.docx]
